# Plasma Hsp90 levels in patients with systemic sclerosis and relation to lung and skin involvement: a cross-sectional and longitudinal study

**DOI:** 10.1038/s41598-020-79139-8

**Published:** 2021-01-07

**Authors:** Hana Štorkánová, Sabína Oreská, Maja Špiritović, Barbora Heřmánková, Kristýna Bubová, Martin Komarc, Karel Pavelka, Jiří Vencovský, Jörg H. W. Distler, Ladislav Šenolt, Radim Bečvář, Michal Tomčík

**Affiliations:** 1 Institute of Rheumatology, Prague, Czech Republic; 2 Department of Rheumatology, First Faculty of Medicine, Charles University, Prague, Czech Republic; 3 Department of Physiotherapy, Faculty of Physical Education and Sport, Charles University, Prague, Czech Republic; 4 Department of Methodology, Faculty of Physical Education and Sport, Charles University, Prague, Czech Republic; 5 Department of Internal Medicine III and Institute for Clinical Immunology, University of Erlangen-Nuremberg, Erlangen, Germany

**Keywords:** Pathogenesis, Rheumatology, Biomarkers

## Abstract

Our previous study demonstrated increased expression of Heat shock protein (Hsp) 90 in the skin of patients with systemic sclerosis (SSc). We aimed to evaluate plasma Hsp90 in SSc and characterize its association with SSc-related features. Ninety-two SSc patients and 92 age-/sex-matched healthy controls were recruited for the cross-sectional analysis. The longitudinal analysis comprised 30 patients with SSc associated interstitial lung disease (ILD) routinely treated with cyclophosphamide. Hsp90 was increased in SSc compared to healthy controls. Hsp90 correlated positively with C-reactive protein and negatively with pulmonary function tests: forced vital capacity and diffusing capacity for carbon monoxide (DLCO). In patients with diffuse cutaneous (dc) SSc, Hsp90 positively correlated with the modified Rodnan skin score. In SSc-ILD patients treated with cyclophosphamide, no differences in Hsp90 were found between baseline and after 1, 6, or 12 months of therapy. However, baseline Hsp90 predicts the 12-month change in DLCO. This study shows that Hsp90 plasma levels are increased in SSc patients compared to age-/sex-matched healthy controls. Elevated Hsp90 in SSc is associated with increased inflammatory activity, worse lung functions, and in dcSSc, with the extent of skin involvement. Baseline plasma Hsp90 predicts the 12-month change in DLCO in SSc-ILD patients treated with cyclophosphamide.

## Introduction

Systemic sclerosis (scleroderma, SSc) is an autoimmune connective tissue disease characterized by vasculopathy and fibrotic changes in multiple organs, particularly the skin and the lungs. It is a rare chronic rheumatic disorder of a complex aetiopathogenesis^1^. Genetic and environmental factors and epigenetics most probably underlie susceptibility to this disease. Scleroderma is traditionally seen as a prototypical multi-system fibrotic disorder mediated by transforming growth factor (TGF)-β and fibroblasts, which produce excessive extracellular matrix proteins such as collagen. However, another hypothesis attributes this disease to the malfunction of the connective tissue repair mechanism in response to injury^1,2^. Patients with SSc can be categorized into two subgroups based on the affected regions: diffuse cutaneous (dc) SSc with proximal involvement, and limited cutaneous (lc) SSc with fibrosis of the skin distal to the elbows or knees, and occasionally of the face and neck^1^. Internal organ complications associated with SSc contribute to its highest morbidity and mortality amongst inflammatory rheumatic disorders^3^. Up to 80% of SSc patients develop interstitial lung disease (ILD), and 25–30% of these patients present with the progressive phenotype. The underlying mechanisms of fibrotic changes in ILD associated with SSc (SSc-ILD) include mesenchymal cell activation, structural changes to epithelial and endothelial cells, and cellular lesions^4^. Currently, ILD is the leading cause of death for SSc patients, accounts for up to 30% of deaths, and has a 10-year mortality of up to 40%^5^. Clinical management of SSc-ILD is challenging and relies on the identification of clinically meaningful disease, assessment of response to pharmacological therapy, and implementation of routine monitoring^3^. Circulating biomarkers reflect relevant physiological or pathological processes, thus may serve as tools for diagnosis, risk evaluation, and prediction of treatment response or safety monitoring. However, disease-specific biomarkers that could be used in routine clinical practice are lacking^6^. Recently, several new promising or effective therapies for SSc-ILD have become available. Therefore, there is an unmet need for disease- or organ-specific biomarkers to assess and to predict treatment response^6,7^.

Heat shock proteins (Hsp) are chaperone proteins first described in 1962 in *Drosophila* based on their increased expression at elevated temperatures^8^. More recent studies found that Hsp are also induced by other forms of cellular stress (e.g. increased pH, disruption of homeostasis, viral infection, reparative healing, and tissue remodeling, etc.)^9,10^. Under physiological conditions as well as during stress response, Hsp recognize and adhere to misfolded proteins in non-native conformations and protect these client proteins against irreversible degradation^11^. In this study, we focused on Hsp90, which is highly conserved and ubiquitously expressed in eukaryotic organisms and in bacteria. It is crucial for many physiological processes, e.g., the cell cycle regulation, cellular apoptosis, protein degradation, and other signalling pathways^12–14^. The abundant Hsp90 activates and stabilizes its various client proteins, such as transcription factors, kinases, and ubiquitin ligases^15^. Some client proteins of Hsp90 are involved in the progression of a diverse group of disorders, e.g., malignancies, neurodegenerative diseases, and bacterial or viral infections^16^. Moreover, Hsp90 is also believed to function as a cytokine in the immune response, and participates in innate and adaptive immunity. Nevertheless, the cytokine-like properties of Hsp90 are yet to be elucidated^17–19^. Of particular interest is the significance of Hsp90 in stabilizing and folding TGF-β receptors and Src kinases that promote fibrosis in SSc^20–22^. Consequently, inhibition of Hsp90 results in proteasomal degradation of TGF-β receptors and Src^23^. We have previously shown that the Hsp90 expression is increased in SSc skin and dermal fibroblasts and is critical for TGF-β signalling. Pharmacological inhibition of Hsp90 effectively blocked the pro-fibrotic effects of TGF-β in cultured fibroblasts and three different preclinical models of SSc^24^. These results have translational implications because several Hsp90 inhibitors have already been used in clinical trials for other indications, mostly tumorous conditions^16,25^.

Therefore, we aimed to assess the systemic levels of Hsp90 in patients with SSc and age-/sex-matched healthy individuals, and analyze its association with SSc-related clinical features with a focus on fibrotic manifestations. Our next aim was to evaluate the potential of systemic Hsp90 as a predictor of response to cyclophosphamide treatment in a group of SSc-ILD patients.

## Results

### Patients and healthy controls: clinical and demographic characteristics

In the cross-sectional analysis, a total of 92 patients and 92 healthy controls were included. Clinical and demographic characteristics of the patients and healthy controls are stated in Table 1.

**Table 1:** Clinical and demographic characteristics of SSc patients and healthy controls: cross-sectional analysis.

Parameters	SSc patients (n = 92)	Healthy controls (n = 92)	p-value
Sex: female/male, n (%)	79 (86)/13 (14)	79 (86)/13 (14)	1.000
Age, median (IQR) (years)	55.0 (45.0–60.5)	51.0 (44.0–61.0)	0.806
Disease duration, median (IQR) (years)	4.0 (1.3–8.0)		
SSc subtype: dcSSc/lcSSc, n (%)	38 (41)/54 (59)		
ANA positivity, n (%)	85 (92)		
ACA positivity, n (%)	16 (17)		
Anti-Scl70 positivity, n (%)	41 (45)		
Raynaud’s phenomenon, n (%)	91 (99)		
Gastrointestinal involvement, n (%)	55 (60)		
Arthralgia and/or arthritis, n (%)	33 (30)		
Interstitial lung disease, n (%)	58 (63)		
Pulmonary arterial hypertension, n (%)	19 (21)		
Cardiac involvement, n (%)	16 (17)		
Renal involvement, n (%)	3 (3)		
Digital ulcers, n (%)	24 (26)		
CRP, median (IQR) (mg/L)	3.2 (1.6–6.5)		
ESR, median (IQR) (mm/h)	13.5 (9.0–28.0)		
mRSS median (IQR)	10.0 (5.0–19.0)		
ESSG activity score median (IQR)	3.3 (2.0–4.0)		
FVC, median (IQR) % predicted	76.0 (62.8–100.4)		
FEV1, median (IQR) % predicted	72.4 (59.9–94.5)		
DLCO, median (IQR) % predicted	61.5 (46.2–75.5)		
SpO_2_, median (IQR) %	96.0 (94.0–97.0)		
Current treatment: GC/MTX/CPA/AZA/MMF, n (%)	48 (44)/10 (9)/4 (4)/4 (4)/1 (1)		

*SSc* systemic sclerosis, *lcSSc* limited cutaneous SSc, *dcSSc* diffuse cutaneous SSc, *IQR* interquartile range, *ANA* antinuclear antibodies, *Anti-Scl-70* anti–DNA-topoisomerase I antibodies, *ACA* anticentromere antibodies, *ESSG* European Scleroderma Study Group, *mRSS* modified Rodnan skin score, *CRP* C-reactive protein, *ESR* erythrocyte sedimentation rate, *FVC* forced vital capacity, *FEV1* forced expiratory volume in 1 s, *DLCO* diffusing capacity for carbon monoxide, *SpO*_*2*_ blood oxygen saturation level, *GC* low dose glucocorticoids (i.e. ≤ 10 mg/day of prednisone), *MTX* methotrexate, *CPA* cyclophosphamide, *AZA* azathioprine, *MMF* mycophenolate mofetil.

In the longitudinal analysis, a total of 30 patients with SSc-ILD with active alveolitis without pulmonary arterial hypertension who underwent a routine 6-month (n = 16) or 12-month (n = 14) treatment with i.v. cyclophosphamide were included. Clinical and demographic attributes of SSc-ILD patients treated with cyclophosphamide are presented in Table 2. The lung function tests FVC, and FEV1 in these SSc-ILD patients remained stable for both 6 and 12 months upon treatment with cyclophosphamide; no statistical differences from baseline were found. However, a trend towards improvement in DLCO from baseline was found after 12 months (Table 2).

**Table 2:** Baseline clinical parameters of patients with SSc-ILD treated with cyclophosphamide: longitudinal analysis.

Parameters	Baseline (month 0) (n = 30)	Month 1 (n = 30)	Month 6 (n = 30)	Month 12 (n = 30)	p-value (p^0–1^; p^0–6^; p^0–12^)
Sex: female/male, n (%)	23 (77)/7 (23)				
Age, median (IQR) (years)	57.7 (46.0–66.0)				
Disease duration, median (IQR) (years)	2.8 (0.9–6.0)				
SSc subtype: dcSSc/lcSSc, n (%)	14 (47)/16 (53)				
ANA positivity, n (%)	25 (83)				
ACA positivity, n (%)	2 (6)				
Anti-Scl70 positivity (%)	16 (53)				
Raynaud’s phenomenon, n (%)	29 (97)				
GI, n (%)	16 (53)				
Arthralgia and/or arthritis, n (%)	11 (37)				
ILD, n (%)	30 (100)				
PAH, n (%)	0 (0)				
CI, n (%)	3 (10)				
RI, n (%)	1 (3)				
Digital ulcers, n (%)	6 (20)				
mRSS median (IQR)	12.0 (4.0–19.0)				
ESSG activity score median (IQR)	2.5 (1.0–4.0)				
Hsp90, median (IQR) (ng/mL)	13.9 (8.6–20.6)	18.0 (9.5–23.3)	12.0 (8.7–21.3)	12.5 (8.9–16.7)^a^	0.683; 0.827; 0.285
CRP, median (IQR) (mg/L)	6.2 (2.7–13.4)	6.7 (2.8–12.4)	5.6 (2.5–14.5)	4.7 (2.2–18.7)	0.564; 0.117; 1.000
ESR, median (IQR) (mm/h)	14.0 (9.5–22.3)	15.5 (10.8–25.8)	14.5 (11.3–23.8)	14.0 (10.0–21.0)	0.275; 0.394; 0.467
FVC, median (IQR) % predicted	76.0 (62.8–100.4)		74.5 (63.0–102.2)	74.4 (60.0–98.3)	–; 0.532; 0.346
FEV1, median (IQR) % predicted	72.4 (59.9–94.5)		76.0 (68.0–96.5)	69.0 (64.5–93.0)	–; 0.532; 0.467
DLCO, median (IQR) % predicted	61.5 (46.2–75.5)		60.0 (47.0–79.0)	64.5 (55.5–79.0)	–; 0.297; 0.090
Current treatment: GC, n (%)	16 (53)				

*SSc* systemic sclerosis, *lcSSc* limited cutaneous SSc, *dcSSc* diffuse cutaneous SSc, *IQR* interquartile range, *ANA* antinuclear antibodies, *Anti-Scl-70* anti–DNA-topoisomerase I antibodies, *ACA* anticentromere antibodies, *GI* gastrointerstinal ivolvement, *ILD* interstitial lung disease, *PAH* pulmonary arterial hypertension, *CI* cardiac involvement, *RI* renal involvement, *ESSG* European Scleroderma Study Group, *mRSS* modified Rodnan skin score, *CRP* C-reactive protein, *ESR* erythrocyte sedimentation rate, *FVC* forced vital capacity, *FEV1* forced expiratory volume in one second, *DLCO* diffusing capacity for carbon monoxide, *GC* low dose glucocorticoids (i.e. ≤ 10 mg/day of prednisone), *p*^*0–1*^ difference between month 0 and 1, *p*^*0–6*^ difference between month 0 and 6, *p*^*0–12*^ difference between month 0 and 12 tested by Friedman's test (for all comparisons).

^a^Values for plasma Hsp90 at month 12 are available only for 14 patients who underwent a 12-month cyclophosphamide treatment.

### Plasma Hsp90 in patients with systemic sclerosis and healthy controls: a cross-sectional analysis

**Figure 1:**
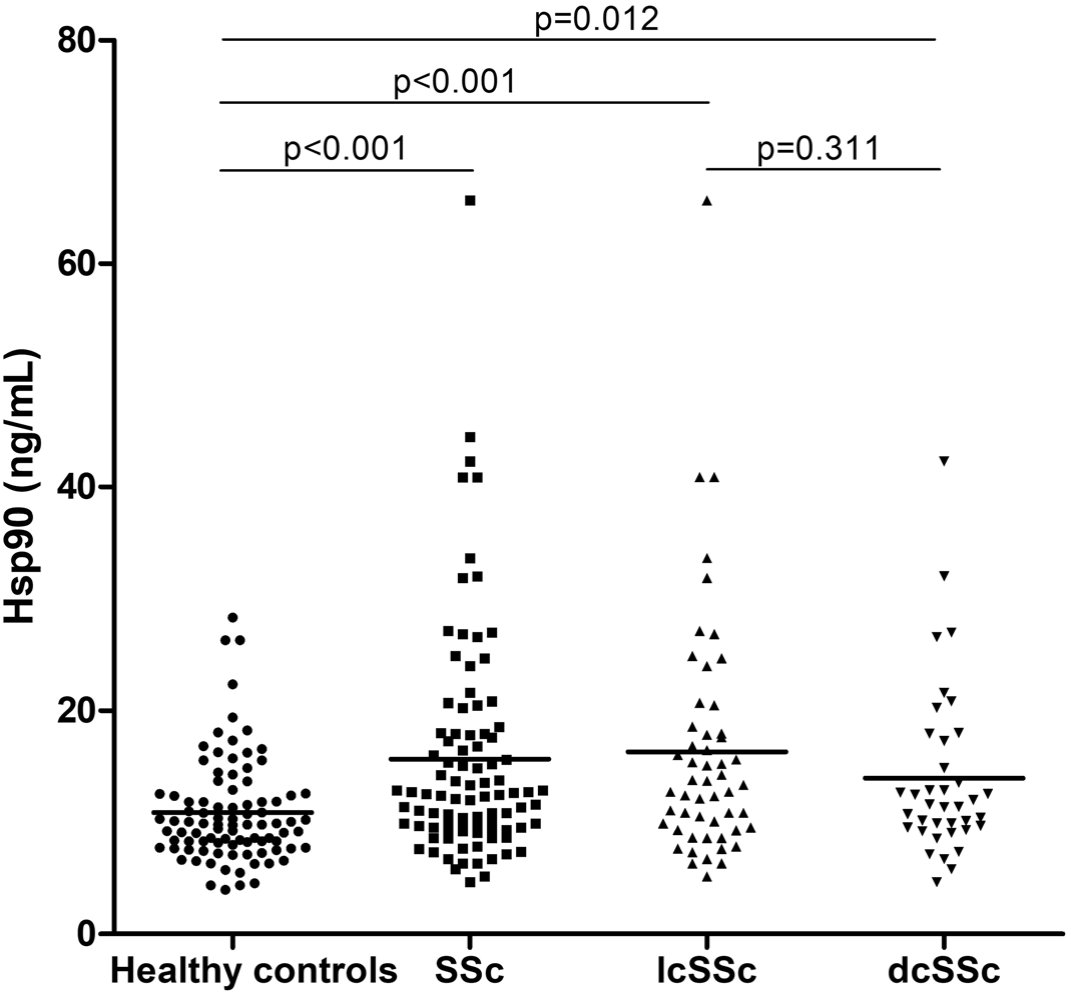
Plasma levels of Hsp90 are significantly elevated in SSc patients and both subsets of SSc (lcSSc and dcSSc) compared with healthy controls. The levels of plasma Hsp90 are comparable in lcSSc and dcSSc patients. Horizontal bars represent the median.

The plasma levels of Hsp90 were elevated in all SSc patients compared to healthy individuals [12.5 (9.6–17.9) vs 9.8 (7.7–12.4) ng/mL, p < 0.001]. After dividing the patients into two basic SSc subtypes, patients with lcSSc [13.4 (9.6–17.9) ng/mL, p < 0.001], as well as patients with dcSSc [11.4 (9.6–17.3) ng/mL, p = 0.012] showed a significant increase in Hsp90 compared to healthy controls. However, no significant difference between lcSSc and dcSSc was detected (p = 0.311) (Fig. 1).

### Hsp90 plasma levels in relation to clinical parameters of SSc: a cross-sectional analysis

**Figure 2:**
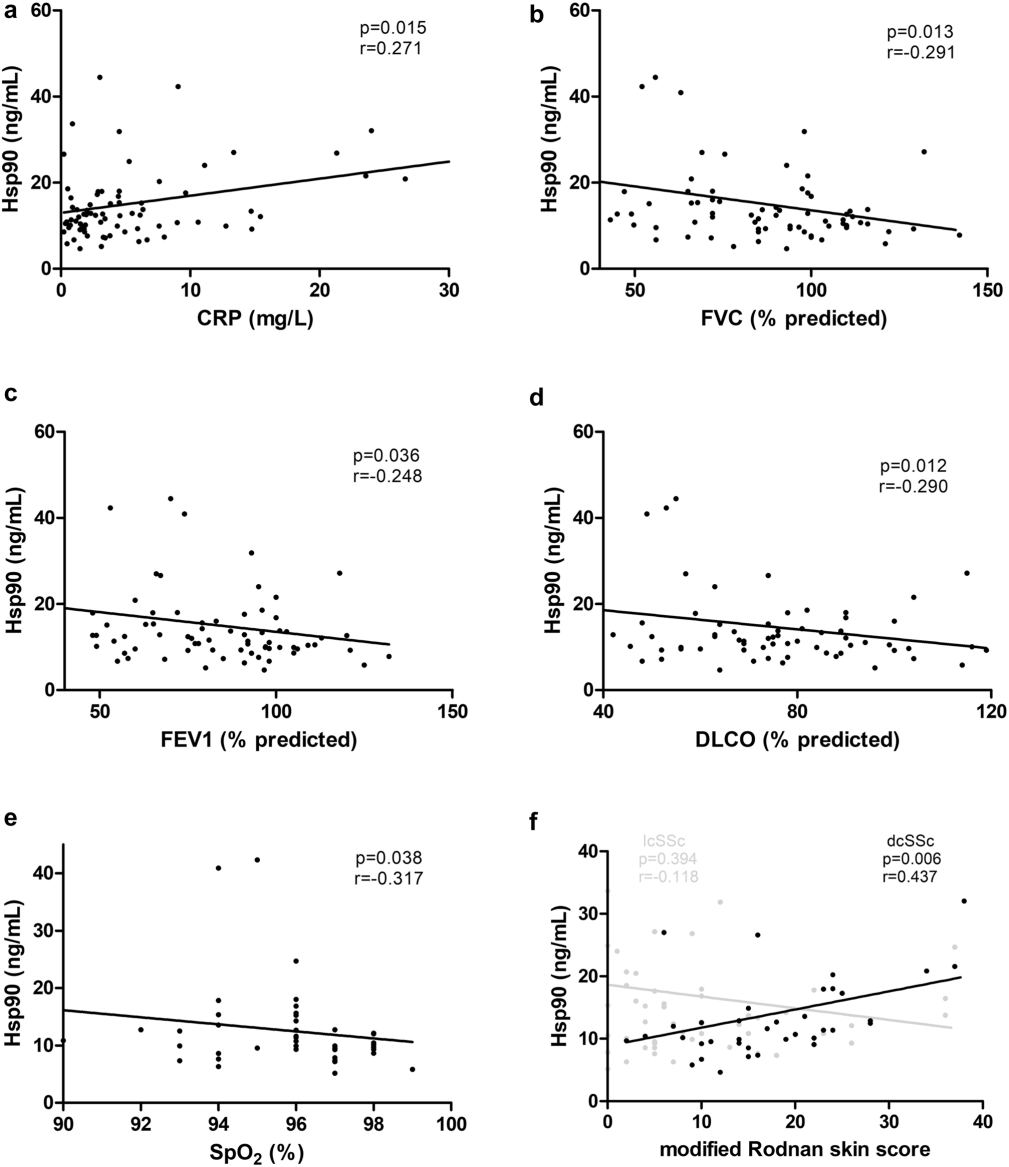
Bivariate correlations of Hsp90 with systemic inflammation, lung function tests, and skin involvement. In all SSc patients, plasma Hsp90 positively correlated with C-reactive protein (CRP) (**a**), and negatively correlated with lung function parameters, such as forced vital capacity (FVC) (**b**), forced expiratory volume in one second (FEV1) (**c**), diffusing lung capacity for carbon monoxide (DLCO) (**d**), and oxygen saturation assessed by pulse oximetry (SpO_2_) (**e**). In patients with diffuse cutaneous (dc)SSc, plasma Hsp90 positively correlated with the extent and severity of skin involvement (black dots), whereas in limited cutaneous (lc)SSc, no significant correlation was found (grey dots) (**f**).

In all SSc patients, plasma levels of Hsp90 positively correlated with C-reactive protein (CRP) (Fig. 2a). Furthermore, extracellular Hsp90 concentrations were increased in patients with deteriorated lung functions: a negative correlation with individual functional parameters of SSc-ILD was detected, such as FVC, FEV1, DLCO, and SpO_2_ (Fig. 2b–e). When adjusted for CRP, correlations with FVC, DLCO, and SpO_2_ (in decreasing order) remained significant in multivariate analysis (Table 3). Of particular interest, only in patients with dcSSc, Hsp90 plasma levels positively correlated with the modified Rodnan skin score (mRSS) (Fig. 2f). Concentrations of extracellular Hsp90 were not significantly affected by other main clinical parameters of SSc (data not shown).

**Table 3:** Univariate and multivariate regression analysis predicting plasma Hsp90 levels based on lung function parameters, unadjusted and adjusted for CRP levels.

Parameter	Unadjusted		Adjusted for CRP	
	b (95% CI)	p-value	b (95% CI)	p-value
FVC	− 0.14 (− 0.23; − 0.04)	**0.005**	− 0.12 (− 0.22; − 0.03)	**0.012**
FEV1	− 0.11 (− 0.22; − 0.01)	**0.043**	− 0.10 (− 0.21; 0.01)	0.079
DLCO	− 0.12 (− 0.22; − 0.02)	**0.017**	− 0.12 (− 0.22; − 0.02)	**0.020**
SpO_2_	− 1.15 (− 2.29; − 0.02)	**0.047**	− 1.13 (− 2.25; − 0.01)	**0.049**

*FVC* forced vital capacity, *FEV1* forced expiratory volume in one second, *DLCO* diffusing capacity for carbon monoxide, *SpO*_*2*_ oxygen saturation assessed by pulse oximetry, *CRP* C-reactive protein, *b* y intercept of a line, *CI* confidence interval. Statistically significant differences (p < 0.05) are marked in bold.

### Plasma levels of Hsp90 in patients with SSc-ILD treated with cyclophosphamide: a longitudinal analysis

**Figure 3:**
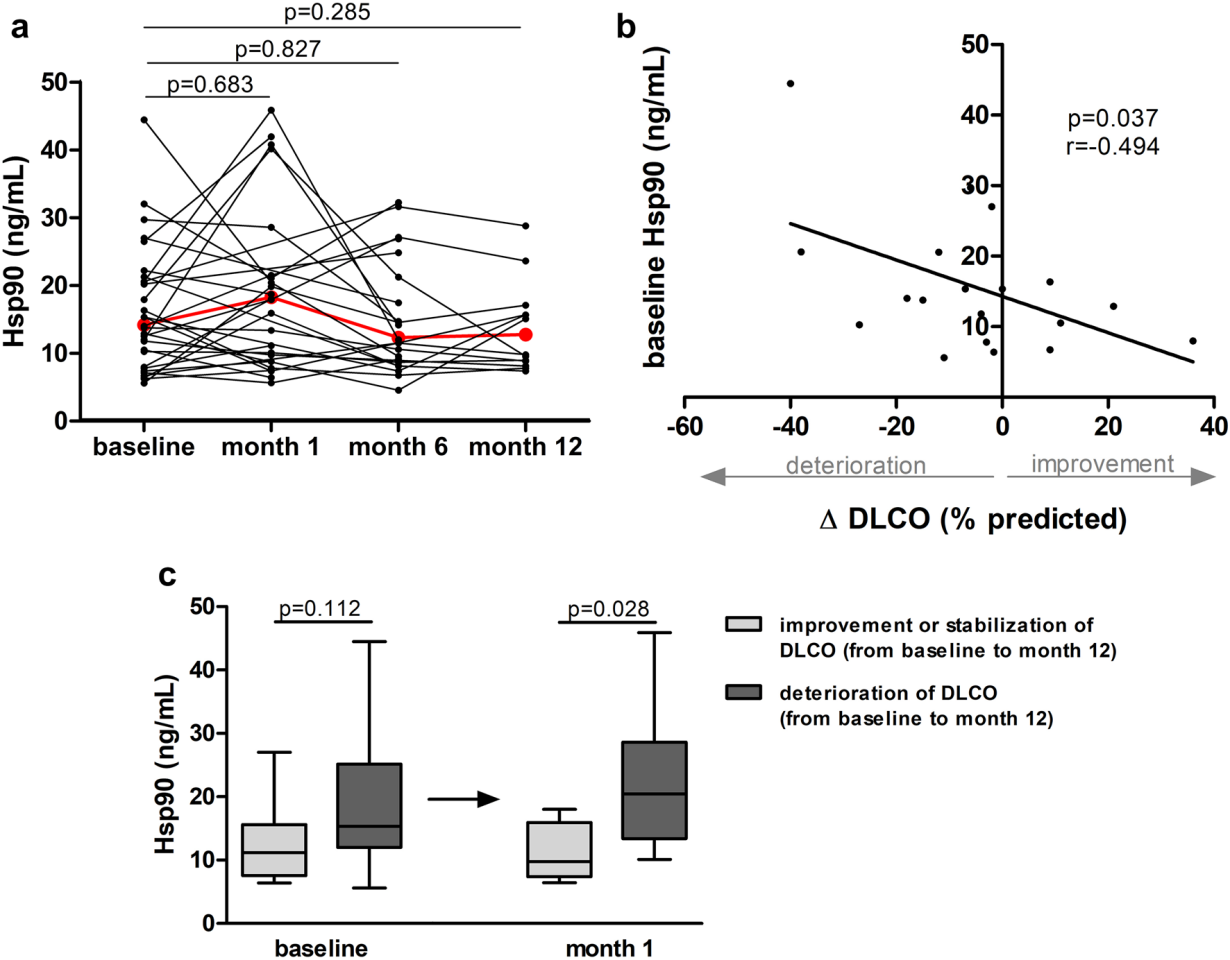
Longitudinal analysis of Hsp90 plasma levels in patients with SSc-ILD treated with cyclophosphamide. (**a**) No significant differences in plasma Hsp90 were detected between baseline and after 1, 6, and 12 months of therapy with cyclophosphamide in patients with systemic sclerosis-associated interstitial lung disease (SSc-ILD). The red line represents the median. Friedman's test was used for all comparisons. (**b**) Lower baseline levels of plasma Hsp90 were significantly associated with larger improvement in diffusing lung capacity for carbon monoxide at month 12 from baseline (ΔDLCO% predicted) upon cyclophosphamide treatment. (**c**) Differences in plasma Hsp90 levels at baseline and one month thereafter in patients grouped according to the course of pulmonary function during the observation time, classified by deterioration and stabilization or improvement of DLCO% predicted at month 12 with respect to baseline. Descriptions of deterioration, stabilization, and improvement are stated in the text. Values are presented as box plots: the boxes represent the 25th–75th percentiles, the lines within the boxes represent the median, and the whiskers represent minimum to maximum.

In the prospective analysis of patients with SSc-ILD treated with 6 or 12 monthly i.v. pulses of cyclophosphamide we did not observe any significant differences between baseline Hsp90 and after 1, 6, and 12 months (Fig. 3a). However, baseline Hsp90 was able to predict the 12-month absolute change in DLCO% predicted upon cyclophosphamide treatment (DLCO_month12–month0_; r = -0.494, p = 0.037) (Fig. 3b). Moreover, plasma Hsp90 levels at month 1 predicted the deterioration (absolute decrease by ≥ 5%), or stabilization (absolute increase or decrease by < 5%) and improvement (absolute increase by ≥ 5%) of DLCO % predicted at month 12 with respect to baseline (Fig. 3c).

## Discussion

In this study, we determined the concentration of plasma Hsp90 in a cross-sectional cohort of patients with SSc compared to healthy controls and analyzed its possible association with disease-specific features. Furthermore, we evaluated the association between plasma Hsp90 and treatment response to cyclophosphamide in a longitudinal cohort of SSc-ILD patients.

For the first time, we report a modest increase in plasma levels of Hsp90 in SSc patients compared to healthy controls, which was statistically significant. However, no significant differences between diffuse and limited cutaneous SSc were observed. To date, systemic levels of Hsp90 have been demonstrated to be elevated in some rheumatic conditions (e.g., systemic lupus erythematosus, axSpA and inflammatory myopathies), as well as in type 1 diabetes and several tumorous conditions (e.g., melanoma, liver and lung cancer)^26–32^.

Our analysis demonstrated a positive correlation between Hsp90 and C-reactive protein (CRP). IL-6 and CRP play an important role in both the pathogenesis and clinical manifestations of SSc and may be useful indicators of disease activity, severity, and poor prognosis^33^. Hsp90 mediates a nuclear factor kappa-light-chain-enhancer of activated B cells (NF-κB) dependent inflammatory pathway associated with increased expression and secretion of IL-6^18,19^. Thus, the positive correlation between Hsp90 and CRP could reflect the possible interaction between Hsp90 and IL-6, which induces CRP production.

Furthermore, increased Hsp90 was associated with impaired lung function, indicated by a decrease in FVC, FEV1, DLCO, and SpO_2_. All parameters, except for FEV1, remained statistically significantly associated with Hsp90 in a multivariate regression model adjusted for CRP. Recently, Sontake et al. demonstrated increased Hsp90 expression in lung fibrosis and elevated Hsp90 ATPase activity in fibroblasts isolated from fibrotic lung lesions. Moreover, inhibition of Hsp90 exerted potent anti-fibrotic effects both in vitro and in murine models of pulmonary fibrosis^34,35^. In addition, Hsp90 could also contribute to the development of lung fibrosis through IL-6. Interleukin-6 signaling is necessary for collagen production and substantially depends on the proper function of the transcription factor Signal Transducer and Activator of Transcription (STAT)3^36–39^. Hsp90 stabilizes and activates STAT3, thus may play a crucial role in the progression of lung fibrosis mediated by IL-6^40^. In our longitudinal analysis of SSc-ILD patients treated with i.v. cyclophosphamide, we did not find any statistically significant changes in the lung function tests or in plasma Hsp90 levels. We observed stabilization of FVC, and a trend towards improvement in DLCO at month 12 from baseline, which are consistent with other observational studies on cyclophosphamide^41^. Nevertheless, baseline plasma Hsp90 was able to predict a 12-month absolute change in DLCO% predicted. In line with this finding, plasma Hsp90 measured after one month of cyclophosphamide treatment predicted the deterioration, stabilization, or improvement of DLCO at month 12. Since there are a few treatment options currently available for SSc-ILD, and novel therapeutic targets are currently under evaluation in ongoing clinical trials, these results might have practical implications in the stratification of SSc-ILD patients according to potential therapeutic response.

In our cross-sectional analysis, we also established a positive correlation between Hsp90 and the extent and severity of skin involvement. However, this was only found in patients with dcSSc, possibly due to more extensive and severe skin involvement compared to lcSSc. This is in line with our previous findings demonstrating increased local expression of Hsp90 in the involved skin and cultured fibroblasts of patients with dcSSc, as well as in the fibrotic skin of three preclinical models of SSc. In that study, inhibition of Hsp90 indicated potent anti-fibrotic properties in three murine models of dermal fibrosis, which mimic different clinical stages of SSc^24^. Moreover, a recent study showed that Hsp90 interacts very closely with fibronectin and may regulate matrix assembly, which is upregulated in SSc^2,42^.

Our study is limited by small numbers, especially in the longitudinal analysis, and the lack of other longitudinal SSc-ILD cohorts treated with other currently available pharmacological agents. Therefore, further studies are needed to confirm and validate our data. However, based on our results, extracellular Hsp90 could become an attractive soluble biomarker of skin and lung involvement in SSc with the potential to predict treatment response in SSc-ILD patients.

## Conclusion

Our study demonstrates that Hsp90 is elevated in the peripheral blood of patients with systemic sclerosis compared to healthy individuals. Plasma Hsp90 levels in SSc patients rise with decreased pulmonary function and increased systemic inflammation. In diffuse cutaneous SSc, Hsp90 is associated with more extensive skin involvement. Of particular interest, in SSc-ILD patients, baseline Hsp90 predicts the 12-month change in DLCO upon cyclophosphamide treatment. These data further support the important role of Hsp90 as a regulator of fibroblast activation and tissue fibrosis in SSc and might have a direct practical application in the real-life stratification of patients with SSc-ILD according to potential therapeutic response.

## Methods

### Patients and healthy controls

All patients involved in this study fulfilled the ACR/EULAR classification criteria for SSc^43^. Plasma samples from all patients and healthy individuals were prepared from the whole blood collected into commercially available EDTA-treated tubes. For the cross-sectional analysis, plasma samples were obtained from 92 Caucasian patients with systemic sclerosis (SSc) and 92 age- and sex-matched Caucasian healthy controls. For the longitudinal analysis, plasma samples were obtained prospectively at baseline, and 1, 6, and 12 months thereafter from 30 Caucasian patients with SSc-ILD with active alveolitis without pulmonary arterial hypertension who underwent a routine 6-month (n = 16) or 12-month (n = 14) treatment with i.v. cyclophosphamide (CPA, 500 mg/m^2^ monthly). Active alveolitis was defined as the presence of areas of ground-glass attenuation on high-resolution computed tomography (HRCT) and reduced levels of diffusing lung capacity for carbon monoxide (DLCO) and/or forced vital capacity (FVC), as described elsewhere^44^. Other SSc-related clinical features were assessed according to generally accepted definitions and recorded, such as the presence of pulmonary arterial hypertension, renal, cardiac and gastrointestinal involvement, Raynaud's phenomenon, and digital ulcers^45^. Skin involvement was evaluated using the modified Rodnan skin score (mRSS)^46^. Disease activity was determined by the European Scleroderma Study Group (ESSG) SSc activity score^47^. Pulmonary function tests (PFT) were routinely performed using standard methods, in accordance with the ATS recommendations^48^. The DLCO was measured by a single-breath method using a gas mixture of 0.2% CO and 8% helium, with correction for hemoglobin. Peripheral oxygen saturation (SpO_2_) was measured by a handheld pulse oximeter (CR-100, Noramedica, Czech Republic). In the longitudinal analysis PFTs were performed at baseline, and 6 and 12 months thereafter, and the results are expressed as a percentage of the normal predicted values based on the patient’s sex, age, and height. The research was confirmed by the local ethics committee at the Institute of Rheumatology in Prague, and each patient signed an informed consent form. All methods were performed in accordance with the relevant guidelines and regulations.

### Laboratory measurements

All samples were stored at − 80 °C until use. Serum levels of C-reactive protein (CRP) were determined by an immuno-turbidimetric technique using an Olympus AU 400 biochemical analyzer (Olympus Optical, Tokyo, Japan), and erythrocyte sedimentation rate (ESR) was measured according to the Fahreus and Westergren method. ANAs were detected using indirect immunofluorescence on HEP2 cells, and the autoantibodies of the ENA complex (anti-U1RNP, anti-Ro, anti-La, anti-DNA-topoisomerase I, anti-Jo-1, anti-P protein, anti-Sm, and anti-centromere) were assayed by immunoblot. Plasma levels of Hsp90 were assessed by a high-sensitivity ELISA kit (eBioscience, Vienna, Austria) according to the manufacturer's protocol. The assay recognizes human Hsp90 alpha. The calculated sensitivity is 0.03 ng/mL. The absorbance value was established at 450 nm by an ELISA reader (SUNRISE; Tecan, Grödig, Austria).

### Statistical analysis

Basic descriptive statistics (mean, median, standard deviation, interquartile range, skewness, and kurtosis) were computed for all variables, which were subsequently tested for normality using the Kolmogorov–Smirnov and Shapiro–Wilk tests. Differences in interval variables (e.g. Hsp90, age, etc.) were tested using the Mann–Whitney U test, while the chi-square test was used to compare frequency counts of categorical variables (e.g. gender). The bivariate relationships between variables under study were assessed using the Spearman correlation coefficient. Linear regression analysis was used to predict patients' Hsp90 levels by a set of predictors (FEV1, FVC, DLCO, SpO_2_) while adjusting for a confounder (CRP). Friedman's test was used to analyze repeated longitudinal measurements taken: (a) at baseline, (b) after 1 month, (c) after 6 months, and (d) after 12 months of therapy with cyclophosphamide. Data are presented as median (IQR) unless stated otherwise. Statistical significance was set at p < 0.05. All analyses were conducted using SPSS version 25 (SPSS, Inc., Chicago, IL, USA). Graphs were created using GraphPad Prism 5 (version 5.02; GraphPad Software, La Jolla, CA, USA).

### Ethics approval and consent to participate

The study was approved by the Ethics Committee of the Institute of Rheumatology in Prague. All study participants were ≥ 18 years of age, and each of them signed an informed consent form. No individual personal data are included.

## Data Availability

The datasets used and/or analysed during the current study are available from the corresponding author on reasonable request.

## Abbreviations

Hsp90: Heat shock protein 90
SSc: Systemic sclerosis
lcSSc: Limited cutaneous SSc
dcSSc: Diffuse cutaneous SSc
TGF-β: Transforming growth factor β
TβRI and TβRII: Transforming growth factor β receptors
IQR: Interquartile range
ANA: Antinuclear antibodies
Anti-Scl-70: Anti-DNA-topoisomerase I antibodies
ACA: Anticentromere antibodies
ATS: American Thoracic Society
ESSG: European Scleroderma Study Group
mRSS: Modified Rodnan skin score
CRP: C-reactive protein
ESR: Erythrocyte sedimentation rate
HRCT: High-resolution computed tomography
PFT: Pulmonary function tests
FVC: Forced vital capacity
FEV1: Forced expiratory volume in one second
DLCO: Diffusing capacity for carbon monoxide
SpO_2_: Blood oxygen saturation level
GI: Gastrointerstinal ivolvement
ILD: Interstitial lung disease
PAH: Pulmonary arterial hypertension
CI: Cardiac involvement
RI: Renal involvement
GC: Low dose glucocorticoids (i.e. ≤ 10 mg/day of prednisone)
MTX: Methotrexate
CPA: Cyclophosphamide
AZA: Azathioprine
MMF: Mycophenolate mofetil
RA: Rheumatoid arthritis
axSpA: Axial spondyloarthritis
NF-κB: Nuclear factor kappa-light-chain-enhancer of activated B cells
TLR: Toll-like receptor
ERK: Extracellular signal-regulated kinases
IL-6R: Interleukin 6 receptor
STAT: Signal transducer and activator of transcription
